# Markov-Type State Models to Describe Non-Markovian Dynamics

**DOI:** 10.1021/acs.jctc.4c01630

**Published:** 2025-02-26

**Authors:** Sofia Sartore, Franziska Teichmann, Gerhard Stock

**Affiliations:** Biomolecular Dynamics, Institute of Physics, University of Freiburg, 79104 Freiburg, Germany

## Abstract

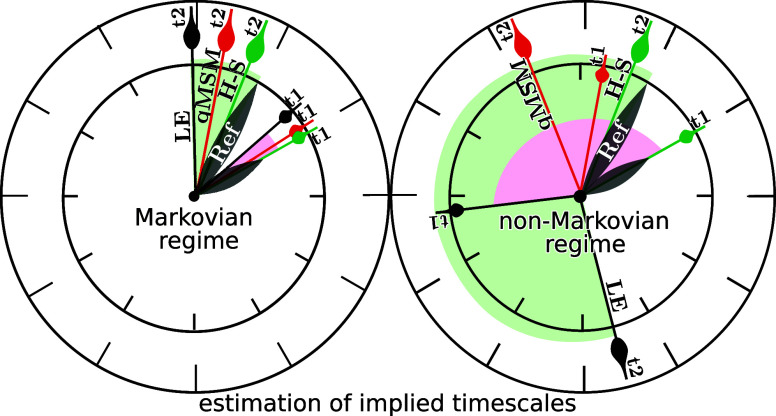

When clustering molecular dynamics (MD) trajectories into a few metastable conformational states, the assumption of time scale separation between fast intrastate fluctuations and rarely occurring interstate transitions is often not valid. Hence, when we construct a Markov state model (MSM) from these states, the naive estimation of the macrostate transition matrix via simply counting transitions between the states may lead to significantly too-short implied time scales and thus to too-fast population decays. In this work, we discuss advanced approaches to estimate the transition matrix. Assuming that Markovianity is at least given at the microstate level, we consider the Laplace-transform-based method by Hummer and Szabo, as well as a direct microstate-to-macrostate projection, which by design yields correct macrostate population dynamics. Alternatively, we study the recently proposed quasi-MSM ansatz of Huang and co-workers to solve a generalized master equation, as well as a hybrid method that employs MD at short times and MSM at long times. Adopting a one-dimensional toy model and an all-atom folding trajectory of HP35, we discuss the virtues and shortcomings of the various approaches.

## 1 Introduction

Classical molecular dynamics (MD) simulations are a versatile tool for gaining insights into complex biomolecular processes.^1^ To facilitate the interpretation of the ever-increasing amount of data obtained from such simulations, coarse grained models such as Langevin equations^2−4^ and Markov state models^5−8^ (MSMs) can be useful. Interpreting MD trajectories in terms of memoryless transitions between metastable conformational states, MSMs are popular because they provide a generally accepted state-of-the-art analysis, promise to predict long-time dynamics from short trajectories, and are straightforward to build using open-source packages.^9−11^ The usual workflow to construct an MSM consists of (i) selection of suitable input coordinates or features, (ii) dimensionality reduction from the high-dimensional feature space to some low-dimensional space of collective variables, (iii) geometrical clustering of these low-dimensional data into microstates, (iv) dynamical clustering of the microstates into metastable conformational states (or macrostates), and (v) estimation of the transition matrix associated with these states.

Here, we are concerned with the last step of the procedure, that is, we want to discuss various approaches (and propose new ways) to estimate the macrostate transition matrix ***T***(*t*). Since these formulations may go beyond the scope of a common MSM, we call them “Markov-type state models”. In what follows, we assume that steps (i) – (iv) resulted in a partitioning of *N* macrostates, such that the MD data can be converted to a state trajectory *I*(*t*). Moreover we assume that all states are connected such that the MSM is ergodic, and that the MD data are time-discrete and represent a stationary and time-homogeneous process (as obtained from a sufficiently sampled equilibrium trajectory).

The macrostate transition matrix ***T***(*t*) contains the probabilities *T*_*IJ*_ that the system jumps from state *J* to state *I* within some prechosen lag time, which can be obtained by simply counting the transitions from *J* to *I* within *t*. When we denote the time-dependent state vector by ***P***(*t*) = (*P*_1_, ···, *P*_*N*_)^*T*^ with state probabilities *P*_*I*_, the time evolution of the state model can be written as
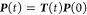
1We note that the time-dependent transition matrix ***T***(*t*) does not involve a dynamical approximation (such as the Markov approximation discussed below), but is directly obtained from the MD data. As a consequence, its practical use is limited by the fact that the calculation of ***T***(*t*) is restricted to times *t* ≲ *t*_max_, where *t*_max_ is the length of the MD trajectories. That is, eq 1 is in essence a state representation of the dynamics, but it does not allow any prediction beyond the time scale of the MD simulations.

The latter can be achieved by invoking a Markov approximation, which assumes a time scale separation between fast intrastate fluctuations and rarely occurring interstate transitions. Assuming that the intrastate fluctuations randomize for times longer than some specific lag time τ, we expect that the transition matrix ***T***(*t*) becomes constant for *t* ≳ τ. Hence, ***T***(*t*) can be approximated by
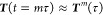
2with *m* = 1, 2, ···, which only requires short MD trajectories (in principle as short as τ) to estimate the transition matrix. Since the time evolution of the MSM is given in steps of τ, the lag time defines the time resolution of the model, and therefore needs to be chosen shorter than the fastest dynamics of interest. This condition, however, is often in conflict with the above requirement of a long enough lag time to achieve Markovianity of the system, i.e., a constant transition matrix. Therefore, in practice one often ends up with a state partitioning that is only approximately Markovian at the desired time resolution, which raises the question of the optimal MSM that best approximates the dynamics in this case.

There are various ways to compute the MSM transition matrix ***T***(τ), in order to optimize the approximation of the true transition matrix ***T***(*t*) in eq 2. For one, we may go beyond the simple counting scheme mentioned above and invoke our knowledge of the microstates the macrostates are built of.^12−15^ This approach exploits that microstates are typically structurally homogeneous, and therefore require only a relatively short lag time to randomize and show Markovian behavior. Macrostates, on the other hand, typically contain numerous structurally different microstates that may be separated by free energy barriers, resulting in long lag times to reach Markovianity. Along these lines, Hummer and Szabo^13^ proposed a valuable formulation to optimally project the microstate dynamics onto the macrostate dynamics, which was shown to achieve significantly improved Markovianity.

Alternatively, we may consider an (in principle) exact equation of motion for the time-dependent state vector ***P***(*t*).^16−20^ By employing projection operator techniques, Zwanzig^16^ derived the generalized master equation
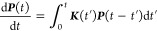
3where ***K***(*t*) is the memory kernel matrix that accounts for the non-Markovian dynamics of the system. To obtain an equation of motion for the transition matrix, we may replace ***P***(*t*) by ***T***(*t*) using eq 1. While the estimation of the memory kernel for (typically noisy and under-sampled) MD data represents a nontrivial task,^21−23^ Huang and co-workers^17^ recently proposed a promising method termed quasi-MSM (qMSM), which was successfully applied to various systems.^24,25^ We also wish to mention a simpler but often effective way of taking memory into account. By “coring” the macrostate trajectory, we either request that a transition from one state to another must reach some predefined core region of the other state,^5,26,27^ or that the system spends a certain minimum time in the new state.^28^ The latter approach, termed “dynamical coring” was shown to considerably improve the Markovianity of the resulting metastable states.^29^

In this work, we discuss the theoretical basis and the performance of various approaches to estimate ***T***(*t*), including the Hummer-Szabo projection,^13^ the qMSM ansatz of Huang and co-workers,^17^ and two new approaches termed “microstate-based” and “hybrid MD/MSM” method, respectively. Adopting a one-dimensional toy model and an all-atom folding trajectory of HP35,^30^ we discuss the applicability as well as the virtues and shortcomings of these approaches.

## 2 Theory and Methods

To estimate the macrostate transition matrix ***T***, we consider three complementary approaches, which use (A) the dynamics of the microstates, (B) the qMSM approximation of the generalized master equation, and (C) a hybrid MD/MSM formulation.

### 2.1 From Micro- to Macrostate Dynamics

In a typical MSM workflow we first construct (say, *n* ∼ 10^2^–10^3^) microstates from the MD data, which are subsequently lumped into a few (say, *N* ≲ 10) macrostates. As explained in the Introduction, we assume that the microstates show Markovian behavior at the chosen lag time τ, which is sufficiently short to resolve the fastest dynamics of interest. Hence, the microstate transition matrix ***t*** = {*t*_*ij*_} satisfies the Chapman-Kolmogorov relation^31^
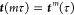
4that is, the microstate MSM with lag time τ reproduces correctly the time evolution of the microstate population vector ***p***(*t*) = (*p*_1_, ···, *p*_*n*_)^*T*^. Here the microstate transition matrix ***t***(τ) is estimated from the MD data, simply by counting the transitions from state *j* to state *i* within lag time τ.

We now combine the above microstates into macrostates *J* = 1, ···, *N* (*N* ≪ *n*), such that each macrostate *J* contains a specific set of microstates {*j*}_*J*_. The macrostate populations *P*_*J*_(*t*) are therefore given by
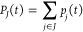
5As for the microstates, we may calculate the corresponding macrostate transition matrix elements *T*_*IJ*_(τ) from the MD macrostate trajectory by counting the transitions from state *J* to state *I* within time τ. Contrary to the microstate MSM, however, the resulting Chapman-Kolmogorov relation for the macrostates is generally not valid, because no clear time separation between intra- and interstate transitions exists. Hence this “local-equilibrium” approximation of the macrostate transition matrix, ***T***_LE_(τ), cannot correctly reproduce the macrostate populations *P*_*J*_(*t*) as found in the MD simulations.

To improve the calculation of the macrostate transition matrix, we follow Hummer and Szabo^13^ and introduce the aggregation matrix ***A*** ∈  with elements *A*_*iJ*_ = 1 (if *i* ∈ *J*) and *A*_*iJ*_ = 0 otherwise. This allows us to rewrite eq 5 in vectorial notation,
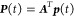
6To derive a similar relation between the *n* × *n* matrix ***t*** and the *N* × *N* matrix ***T***, we define the diagonal matrices ***D***_*n*_ ∈  and ***D***_*N*_ ∈ , whose elements are given by the normalized equilibrium populations  and , respectively.^13^ Using ***D***_*N*_ and ***D***_*n*_ to normalize the aggregation matrices ***A*** and ***A***^*T*^, we find , as can be verified by insertion. This leads to the desired relation between micro- and macrostate transition matrices
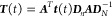
7

It can be evaluated in various ways. In the local-equilibrium approximation, we first calculate the microstate transition matrix ***t***(τ), perform the transformation to get the corresponding macrostate transition matrix ***T***(τ), and arrive at the MSM expression
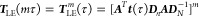
8That is, to evaluate , in effect we only need the definition of the macrostates.

What is more, eq 7 suggest also an alternative and potentially better approximation of ***T***(*t*). Since we initially assumed that microstate transition matrix ***t*** (τ) satisfies the Chapman-Kolmogorov relation in eq 4, we may first calculate the microstate time evolution via eq 4 and subsequently perform the transformation to the macrostate transition matrix, i.e.,
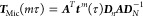
9This microstate-based evaluation of the macrostate transition matrix preserves all dynamical properties established for the microstates. For example, it yields by design exact macrostate population dynamics, and also provides correct state-to-state waiting times and transition pathways from microstate Monte Carlo Markov chain simulations with subsequent projection on the macrostates. While the definition of ***T***_Mic_ is straightforward and its virtues are clearly promising, this microstate-based approach has to the best of our knowledge not yet been mentioned. Compared to the local-equilibrium approximation that yields a constant transition matrix ***T***_LE_(τ) and the MSM relation (eq 8), however, the microstate-based evaluation results in a time-dependent macrostate transition matrix.

Alternatively, Hummer and Szabo^13^ used a Laplace transformation to derive a long-time approximation of the macrostate transition matrix, which leads to an optimal projection of the microstate dynamics onto the macrostate dynamics. They obtained  with
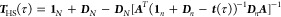
10which provides a constant transition matrix ***T***_HS_(τ), but requires the inversion of *n*- and *N*-dimensional matrices.

Hence, we have introduced three ways to calculate ***T***(*m*τ) from ***t***(τ): The standard local-equilibrium approximation (eq 8), the Hummer-Szabo expression (eq 10), and the microstate-based evaluation (eq 9).

### 2.2 qMSM Evaluation of the Generalized Master Equation

Huang and co-workers^17^ recently derived a generalized master equation for the transition matrix ***T***(*t*) of the form
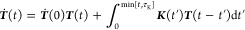
11Compared to eq 3, it contains the additional term ***Ṫ***(0)***T***(*t*), which reflects the time evolution of the system without interaction with the environment. Moreover, the upper bound of the integral assumes that the memory kernel ***K***(*t*) decays within the (presumably short) time τ_K_.

To solve eq 11, we discretize the equation by introducing *t* = *t*_*n*_ = *n*Δ*t*, ***T***(*t*_*n*_) = ***T***_*n*_ and ***K***(*t*_*n*_) = ***K***_*n*_, yielding
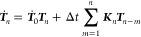
12The main idea of the qMSM approach is to first obtain a short-time approximation of the transition matrix from the MD data, ***T*** ≈ ***T***_MD_. Subsequently, ***T***_MD_ is used to iteratively solve eq 11 for the memory matrix via
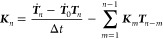
13Inserting the resulting ***K***_*n*_ in eq 12, we obtain the desired evolution of ***T***(*t*) for long times (*t* ≫ τ_K_). Hence the qMSM formulation facilitates a straightforward approximate solution of an in principle exact generalized master equation, and also explicitly provides the memory matrix ***K***(*t*) that accounts for the non-Markovian dynamics of the system. Requiring only MD data of length *t* ≈ τ_K_, qMSM allows us to predict long-time dynamics from short trajectories. The above equations were implemented in an in-house Python code following the well–documented tutorial in ref (32); the kernel decay time τ_K_ was inferred directly from the memory kernel as explained below.

While the qMSM inversion scheme [i.e., solving eq 13 to get ***K*** and inserting it in eq 12 to get ***T***] appears to be a straightforward data-driven approach to parametrize the generalized master equation, there are several peculiarities of the qMSM method. For one, we note that–given long enough MD data– the MD-based matrix ***T***_MD_ is the best transition matrix you can get. Hence, by calculating ***K*** from ***T***_MD_ via eq 13 and inserting it in eq 12, the resulting transition matrix ***T*** can only be worse than the initial matrix ***T***_MD_. Iterating the procedure [by using the new ***T*** to again calculate ***K*** and then again ***T***] does not help either, because we obtain the same result as in the first iteration (as readily shown by insertion).

Another issue is that qMSM keeps the additional term ***Ṫ***(0)***T***(*t*), which typically vanishes for symmetry reasons,^16,33^ but may be nonzero in a numerical evaluation. Interestingly, this term exactly cancels the memory kernel at *t* = 0 [which can be shown by calculating ***T***_*n*_ and ***K***_*n*_ for *n* = 0 from eqs 12 and 13, respectively]. While this is again a consequence of the chosen discretization, it seems odd, because ***K***_0_ is the only nonzero term in the Markov limit. In all cases considered, however, the neglect of the term seems to hardly change the numerical results. Another practical issue is that the calculation of the memory matrix from eq 13 can be seriously plagued by noise resulting from insufficiently sampled MD data and the computation of time derivatives. As a remedy, recently an integrative ansatz^25^ and a time-convolutionless approach^20^ of the generalized master equation were proposed.

### 2.3 Hybrid MD/MSM Formulation

In the Introduction, we opposed in eqs 1 and 2 two standard ways to estimate the macrostate transition matrix ***T***(*t*) = {*T*_*IJ*_(*t*)}. On the one hand, we may use the MD data to directly count the transitions from state *J* to state *I* within time *t*. The resulting transition matrix ***T***_MD_(*t*) generates the correct time evolution of the state model according to eq 1, but is limited to times *t* ≈ *t*_max_ with *t*_max_ being the length of the MD trajectories (and actually even some hundreds MD frames less to facilitate a sufficient time average). On the other hand, we may invoke the Markov approximation to obtain ***T***(*t* = *m*τ) ≈ ***T***^*m*^(τ), which assumes that the transition matrix ***T***(*t*) becomes constant for *t* ≳ τ. While this only requires short MD trajectories, we need to choose a lag time τ that is long enough to achieve Markovianity, but at the same time short enough to resolve the fastest dynamics of interest.

To have the best of both worlds, we may simply combine the two approaches by using ***T***_MD_(*t*) at short times and employing some formulation (including local equilibrium, Hummer-Szabo, or microstate-based) to construct a MSM using the long lag time *t*_max_, i.e.,
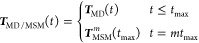
14Hence, we use at short times the approximation-free transition matrix ***T***_MD_(*t*), including full time resolution (as given by the MD data). At long times, the MSM typically can use a rather long lag time, such ensuring Markovianity. In this way, we overcome the restrictions of a common MSM using a compromise for τ, which is too long at short times and too short at long times.

## 3 Results

To compare the performance of the different methods, we test them on two model systems: A one-dimensional toy model lumping four microstates into two macrostates, and a recently established^34^ benchmark MSM of the folding of villin headpiece (aka HP35) using a long MD trajectory by Piana et al.^30^

### 3.1 One-Dimensional Toy Model

**Figure 1:**
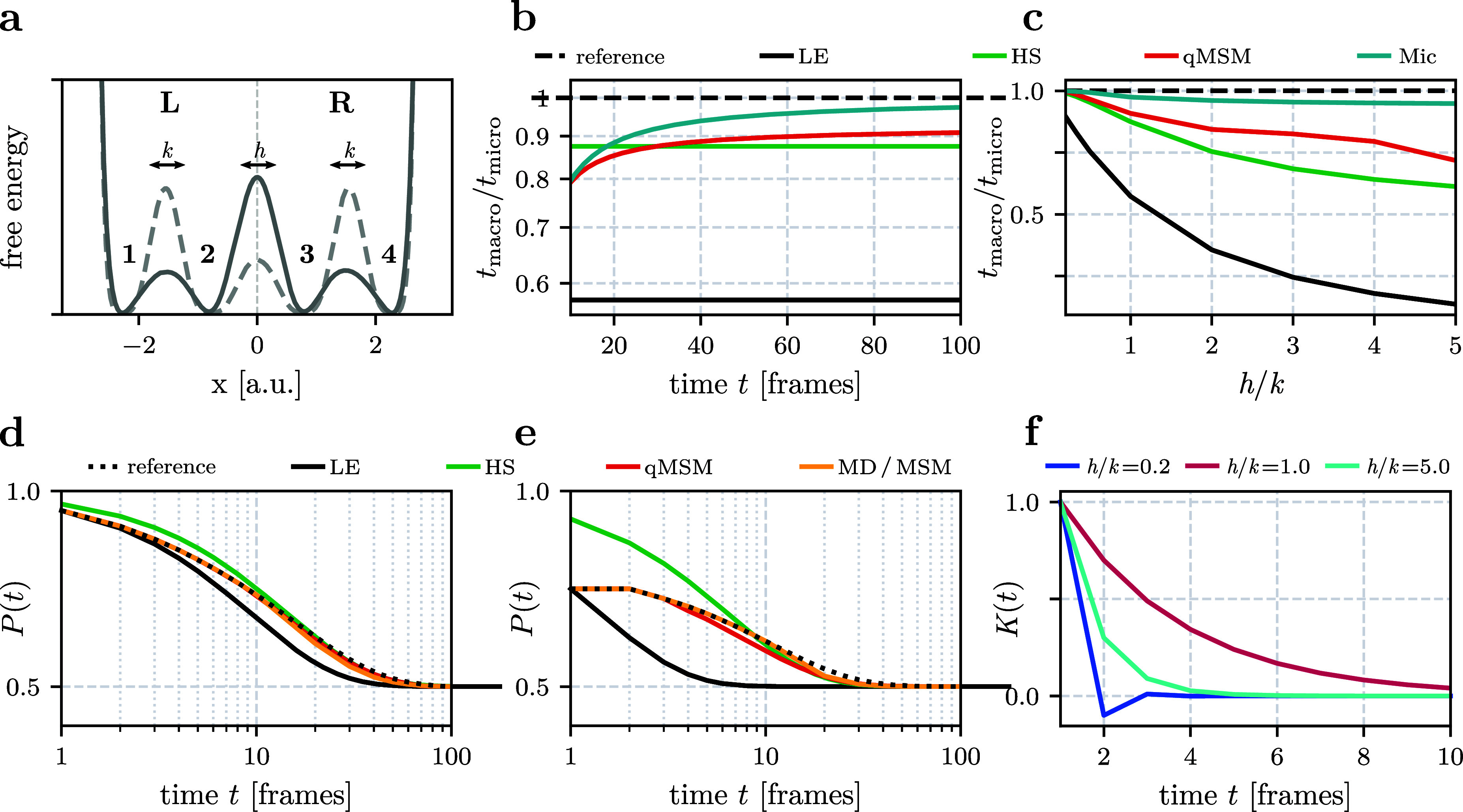
One-dimensional toy model. (a) Schematic free energy landscape, indicating four microstates 1, 2, 3 and 4, which are lumped into two macrostates *L* and *R*. Depending on the ratio of the interstate transition probability *h* and the intrastate transition probability *k*, the macrostate dynamics is Markovian (*h*/*k* ≪ 1, dark gray) or non-Markovian (*h*/*k* ≳ 1, dashed). (b) Ratio of the macroscopic and microscopic implied time scales *t*_macro_/*t*_micro_ of the two-macrostate model for *h*/*k* = 1. Compared are the reference result obtained from the microstates (dashed black), the local equilibrium approximation (black), and the Hummer-Szabo projection (green), as well as the time evolution the result from qMSM (red) and of the microstate-based result (blue). (c) Normalized implied time scales obtained from the various methods, drawn as a function of the Markovianity parameter *h*/*k*. Time evolution of the macrostate population of the various methods, shown for (d) *h*/*k* = 1 and (e) *h*/*k* = 5. Also shown are results of the hybrid MD/MSM calculation (eq 14) with *t*_max_ = 10. (f) Normalized memory kernel *K*(*t*) of the qMSM method, obtained for *h*/*k* = 0.2 (blue), *h*/*k* = 1 (red), and *h*/*k* = 5 (green).

As a simple instructive model, Figure 1a shows the free energy landscape of a system with four microstates *i* (*i* = 1, ···, 4), which are lumped into two macrostates *I* (*I* = *L*, *R* for left and right state). Following Hummer and Szabo,^13^ we define the model via its microstate transitions
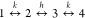
with transition probabilities *k* and *h*. The resulting microstate transition matrix reads
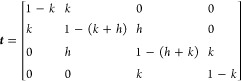
15Because ***t*** is symmetric and detailed balance holds (*t*_*ij*_π_*j*_ = *t*_*ji*_π_*i*_), the microstate equilibrium populations are π_*i*_ = 1/4. The eigenvalues of the matrix are λ_0_ = 1, λ_1/3_ = 1 − *h* − *k* ±  and λ_2_ = 1 − 2*k*. Hence, the longest (nontrivial) microstate implied time scale is
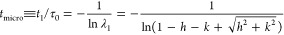
16where we introduced the unit time τ_0_ to define a dimensionless time *t*_micro_.

When we combine microstates 1 and 2 into macrostate *L* and microstates 3 and 4 into macrostate *R*, the probability *k* accounts for intrastate transitions (i.e., within *L* or *R*) and *h* accounts for interstate transitions (i.e., between *L* and *R*) of the macrostate model, see Figure 1a. We study the model both in the case of good lumping (i.e., a sufficient time scales separation between fast intrastate and slow interstate motions) and in the case of bad lumping. The latter means that we have a low energy barrier between macrostates *L* and *R*, and high barriers between microstates that belong to the same macrostate. This leads to a bad time scales separation, causing the model to behave non-Markovian.

Due to the simplicity of the model, we can derive analytical expressions for the 2 × 2 macrostate transition matrix. In the local equilibrium approximation (eq 8), we obtain
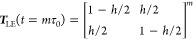
17Notably, ***T***_LE_ does not depend on the intrastate transition rate *k*, as it is assumed to be much higher than any other time scale of the macrostate model. The single (nontrivial) macrostate implied time scale is
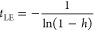
18The Hummer-Szabo projection (eq 10) gives  with^13^
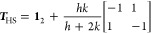
19which takes into account both intra- and interstate transitions. The resulting implied time scale is
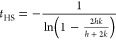
20In the microstate-based approach (eq 9) and the qMSM method (eq 12), the transition matrices ***T***_Mic_(*t*) and ***T***_qMSM_(*t*) are calculated numerically, because no simple analytical expressions exist. Note that the implied time scales of ***T***_Mic_(*t*) and ***T***_qMSM_(*t*) depend on time, while the implied time scales *t*_micro_, *t*_LE_, and *t*_HS_ are constant.

As a first test, we wish to study how the various formulations reproduce the reference implied time scale *t*_micro_ of the microstates. Choosing *k* = *h* = 0.1 (weakly non-Markovian case), Figure 1b shows that the local equilibrium approximation *t*_LE_ underestimates *t*_micro_ almost by a factor 2, while the Hummer-Szabo result is only shorter by 13%. The results obtained for ***T***_Mic_(*t*) and ***T***_qMSM_(*t*) reveal that the microstate-based method converges to the correct result and that qMSM converges to a value that is 9% shorter than *t*_micro_. (In all cases, we choose the qMSM kernel decay time τ_K_ such that the normalized memory kernel satisfies *K*(τ_K_) ≤ 0.1.) To compare to a largely Markovian case (*h*/*k* = 0.2), Figure S1 shows that all methods yield the correct time scale *t*_micro_, except for the local equilibrium approximation, which gives *t*_LE_ = 0.9*t*_micro_.

We now choose *t* = 100 (where qMSM and microstate-based results are virtually constant), and vary the Markovianity parameter *h*/*k* from *h*/*k* ≪ 1 (Markovian case) to *h*/*k* ≳ 1 (non-Markovian case). While for *h* = 0.1, *k* = 0.5 all methods yield the correct microstate time scale *t*_micro_ (except for *t*_LE_), the results vary considerably in the non-Markovian case. Choosing *h* = 0.5, *k* = 0.1, for example, *t*_LE_ underestimates *t*_micro_ by 87%, *t*_HS_ by 40%, and *t*_qMSM_ by 27%, while the microstate-based result closely matches the reference.

We now consider the quality of the underlying time-dependent transition matrices. As the diagonal elements *T*_*II*_(*t*) (*I* = *L*, *R*) represent the state populations *P*_*L*_(*t*) = 1 – *P*_*R*_(*t*), which also determine the off-diagonal elements *T*_*LR*_ = 1 – *P*_*L*_ and *T*_*RL*_ = 1 – *P*_*R*_, it is sufficient to focus on *P*_*L*_(*t*) ≡ *P*(*t*). By design, *P*(0) = 1 and *P*(*∞*) = Π_*L*_ = 0.5, such that the various formulations only differ by the rate of the decay. Because the microstates produce the exact dynamics of the model, the microstate-based calculation *P*_Mic_(*t*) coincides with this reference.

Choosing *h*/*k* = 1 and *h*/*k* = 5, Figure 1d,e compare the population *P*(*t*) obtained from the various formulations. While for *h*/*k* = 1 all methods are quite close to the reference results, we find significant deviations in the strongly non-Markovian case *h*/*k* = 5, where *P*(*t*) in fact reveals two time scales. That is, the population exhibits a strong initial decay to *P*(1) = 0.75 during the first time step, reflecting fast transitions over the shallow barrier at *x* = 0 (Figure 1a). All formulations reproduce this value correctly, except for the long-time approximation *P*_HS_(*t*), which coincides with the reference for *t* ≳ 7. Being a short-time approximation, *P*_LE_(*t*) decays significantly too fast for *t* > 1. The qMSM calculation represents a clear improvement over these approximations and reproduces the exact results closely. Finally, we also show the hybrid calculation (eq 14), which uses until *t*_max_ = 10 the exact transition matrix ***T***_MD_(*t*), and for longer times the local approximation ***T***_LE_(*t* = *mt*_max_). For this choice of *t*_max_, the hybrid result virtually matches the reference at all times.

Unlike the other methods, the generalized master eq 11 underlying the qMSM method formulation involves the calculation of a memory matrix ***K***(*t*), which reports on the non-Markovianity of the dynamics. Since for symmetry reasons *K*_*LL*_ = *K*_*RR*_ = −*K*_*LR*_ = −*K*_*RL*_, it is sufficient to focus on *K*_*LL*_(*t*). Considering the cases *h*/*k* = 0.2, 1, and 5, Figure 1f shows the normalized memory kernel *K*(*t*) = *K*_*LL*_(*t*)/*K*_*LL*_(1). As expected, in the Markovian limit (*h*/*k* = 0.2) the memory decays essentially within a single time step. Less expected, though, we find that the slowest decay (∼2.8) is obtained for weakly non-Markovian case (*h*/*k* = 1), while in the strongly non-Markovian case (*h*/*k* = 5), the decay (∼0.8) is again faster, although clearly slower than the Markovian case.

To explain this finding, we recall that the memory kernel *K*(*t*) is meant to reflect the intrastate dynamics of the macrostates, which in turn depends on the implied time scales *t*_*k*_ (*k* = 1, 2, 3) and associated eigenvectors of the microstate MSM. As shown in Table S1, the first time scale and eigenvector clearly indicate transitions between the left states (*L* = 1, 2) and the right states (R = 3, 4), while the second and third eigenvectors reflect transitions within *L* and *R*. In the Markovian limit (*h*/*k* = 0.2), we find *t*_2_ = 0.45 and *t*_3_ = 0.26, which indeed coincide with the decay of the memory kernel *K*(*t*) in Figure 1d. Moreover, for *h*/*k* = 1, we obtain *t*_2_ = 4.5 and *t*_3_ = 2.4, which again are similar to the decay time (∼2.8) of *K*(*t*). In the strongly non-Markovian case (*h*/*k* = 5), however, this coincidence is less clear, as we get *t*_2_ = 4.5 and *t*_3_ = 0.45, but a memory decay time of 0.8. Interestingly, we also find that for *h*/*k* = 5 the amplitude *K*(1) of the memory is about 20 times larger than in the first two cases. These findings indicate that the interpretation of the qMSM memory kernel in terms of memory time scales is generally not straightforward.^35,36^

### 3.2 Folding of HP35

To learn how the above findings for the toy model generalize to the case of all-atom MD data, we now consider the folding of villin headpiece (HP35) as a well-established model problem. As in previous work,^34,37^ we use a ∼300 μs-long MD trajectory of the fast folding Lys24Nle/Lys29Nle mutant of HP35 at *T* = 360 K by Piana et al.,^30^ which shows about 30 folding events. Following the benchmark study of Nagel et al.,^34^ we employ the following MSM workflow. First, we use MoSAIC correlation analysis^38^ to perform a feature selection resulting in 42 contact distances. Next, we eliminate high-frequency fluctuations of the distance trajectory, by employing a Gaussian low-pass filter^37^ with a standard deviation of σ = 2 ns. Employing principal component analysis on these contacts,^39^ we use the resulting first five components for subsequent robust density based clustering^40^ into 547 microstates. In a final step, we adopt the most probable path algorithm^41^ to dynamically lump the microstates into 12 metastable macrostates, using a lag time of 10 ns. The resulting dynamical model compares favorably to MSMs from alternative combinations of methods^34^ and explains the folding of HP35 as cooperative transition between folded and unfolded energy basins and several intermediate states. In this work, we employ the partitioning of the MD trajectory into micro- and macrostates, in order to calculate the various versions of the macrostate transition matrix introduced above.

**Figure 2:**
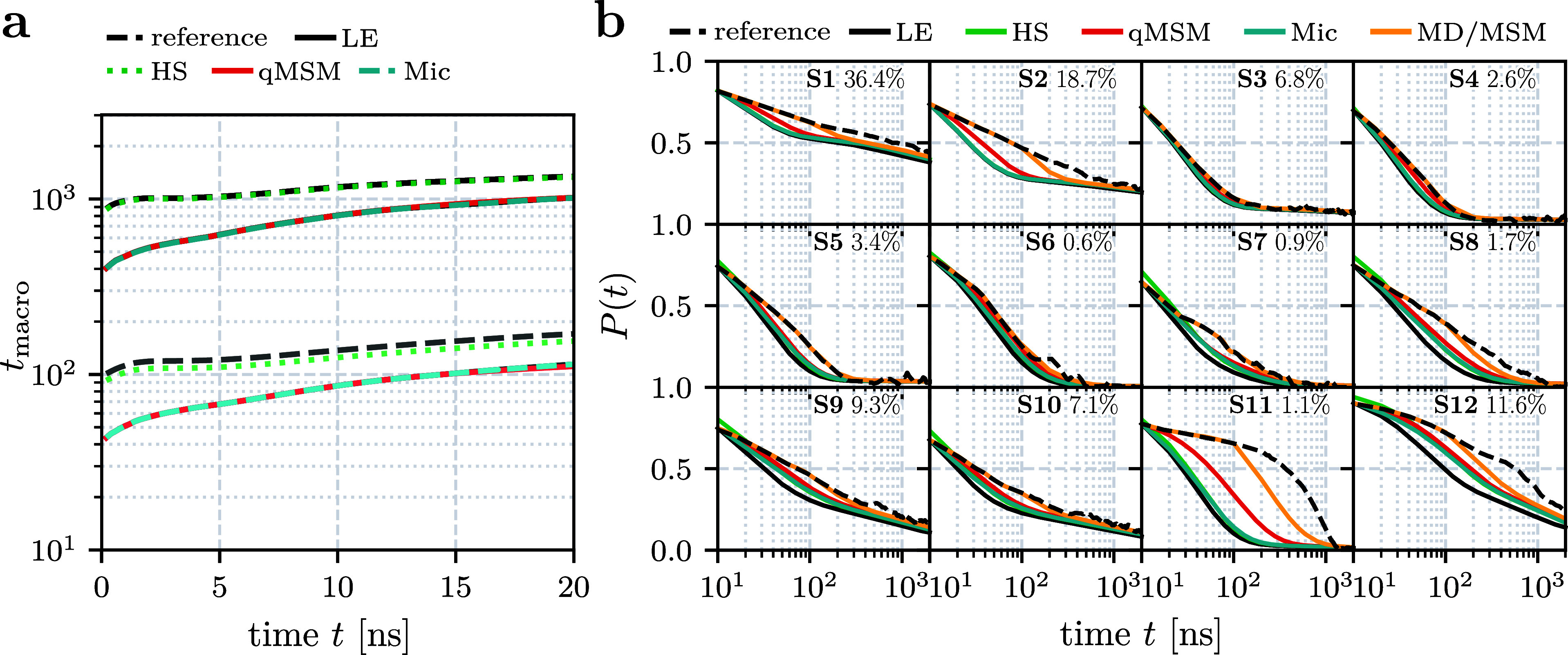
Twelve-state MSM constructed from a 300 μs-long MD trajectory^30^ of the folding of HP35. (a) First two implied time scales obtained from the microstates (dashed black, reference), the local equilibrium approximation (using the lag time τ = 10 ns, black), the Hummer-Szabo projection (using τ = 10 ns, green), the microstate-based result (blue), and the result from qMSM (using the kernel time τ_K_ = 10 ns, red). (b) Chapman-Kolmogorov test of the 12 macrostates, comparing the reference MD state trajectory to the various versions of the theory. Also shown are results of the hybrid MD/MSM calculation (eq 14) with *t*_max_ = 100 ns (yellow).

We begin with the discussion of the first two implied time scales, which are depicted in Figure 2a as a function of the lag time. As discussed in ref (34)., the slowest time scale (∼1.5 μs) corresponds to the overall folding process, while the next two time scales (∼0.1 μs) account for conformational rearrangement in the native and the unfolded basins. Taking again the microstate time scales as a reference, we overall find the Hummer-Szabo results being in excellent agreement, followed by the results from the local equilibrium approximation, which virtually coincide with the qMSM and the microstate-based results.

As most important assessment, Figure 2b shows the Chapman-Kolmogorov test of the 12 macrostates, which compare the macrostate projection of the MD trajectory (providing the reference) to the various versions of the theory. As a result of a careful selection of features and methods, the benchmark model^34^ represents a valuable MSM. Consequently, the deviations of the various methods are overall only minor, and already the simple local equilibrium approximation matches the correct population dynamics of most macrostates quite closely. Quite similar is the Hummer-Szabo projection, despite its superior modeling of the implied time scales above.

Recalling that the microstate-based calculation should coincide with the reference MD results in the case of a truly Markovian partitioning of the microstates, we learn that the microstates are a main reason for the deviations of the Chapman-Kolmogorov tests. Not relying on the quality of the microstates, the qMSM in fact represents an improvement of the microstate-based methods, particularly in the case of ill-defined states such as states 2 and 11. (Judging from the time evolution of the qMSM memory matrix ***K***(*t*) shown in Figure S2, we chose τ_K_ = 10 ns where, apart from residual fluctuations around zero, all elements *K*_*IJ*_(*t*) are safely decayed.) Even better is the performance of a hybrid calculation (eq 14), which uses until *t*_max_ = 100 ns the exact transition matrix ***T***_MD_(*t*), and for longer times the local approximation ***T***_LE_(*t* = *mt*_max_). Apart from the ill-defined states 2 and 11, the hybrid calculation matches the reference very accurately.

While a single, long MD trajectory is certainly perfect for building an MSM, in practice we often have many short trajectories (since they are readily computed in parallel). In principle, trajectories as short as the lag time τ and the kernel time τ_K_ should be already sufficient to construct an MSM and a qMSM, respectively. However, the validity of this presumption has been rarely assessed due to the lack of long reference trajectories. As we used τ = τ_K_ = 10 ns in the calculations above, and we need some extra time for the time averages to calculate transition probabilities, we decided to split the 300 μs trajectory in 10,000 × 30 ns (150 frames) long pieces, such that we can average over 100 frames. Note that 30 ns is quite short considering the overall folding time of ∼2 μs of HP35. As the MSM workflow explained above employs no time information up to the density based clustering included,^42^ we can use the same microstates. On the other hand, the dynamical lumping of these states via the most probable path algorithm^41^ requires calculations based on the transition matrix, which differs in the case of one long and of many short trajectories. Hence, the resulting macrostates differ minor from the ones obtained from the single 300 μs trajectory, see Figure S3. Lastly, we again calculated the macrostate transition matrices for the various theoretical formulations.

**Figure 3:**
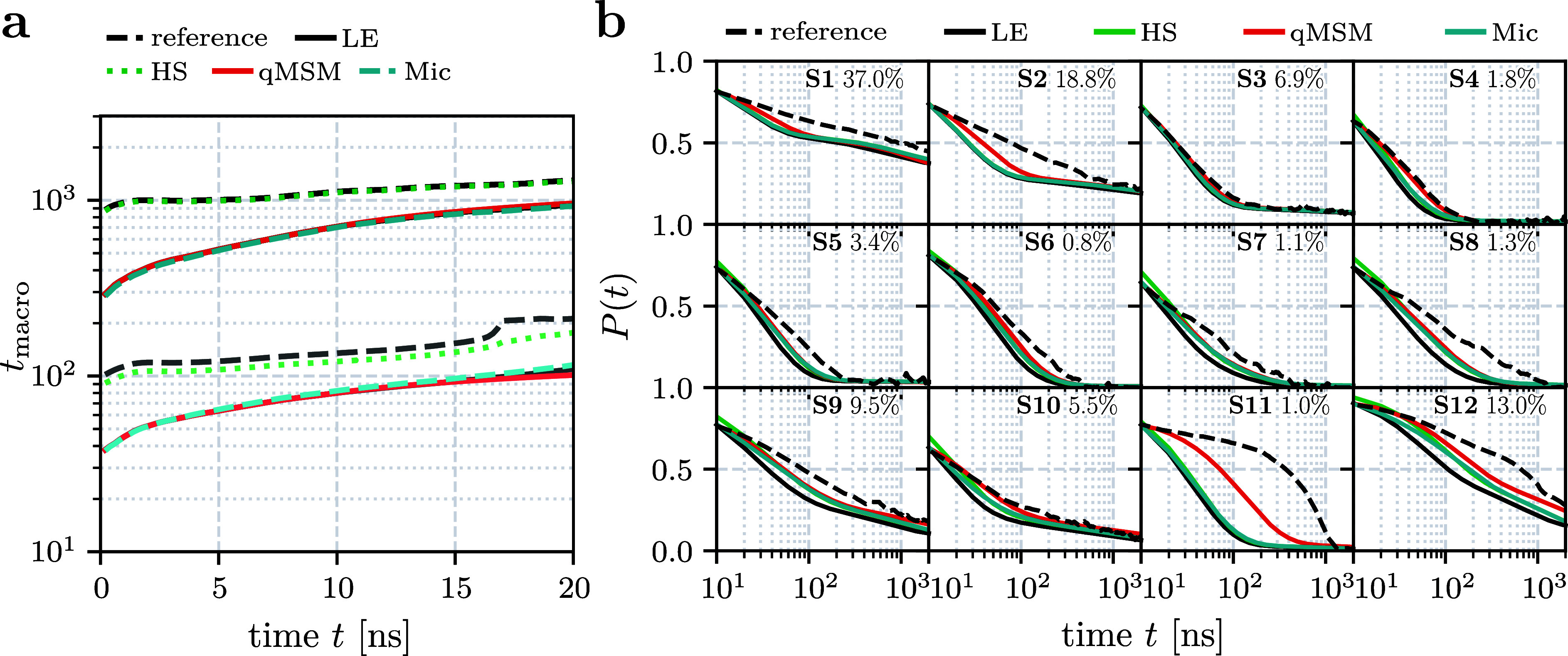
(a,b) As in Figure 2, except that the original 300 μs-long trajectory of HP35 was split in 10,000 × 30 ns trajectories. The hybrid MD/MSM method is not shown, as for *t*_max_ = 10 ns it coincides with the local equilibrium approximation.

Figure 3 shows the implied time scales and Chapman-Kolmogorov tests of the resulting MSMs. Overall the short-trajectories results look very similar to the results from the single long trajectory (Figure 2), thus confirming the promise of MSMs to describe long-time dynamics from short trajectories. However, it should be kept in mind that these short trajectories used adequately chosen initial conditions, because they were taken from a long trajectory that exhibits numerous folding and unfolding transition. This is less obvious without such a reactive trajectory, although various strategies have been proposed to obtain good initial conditions along the reaction paths of a considered process.^43−45^

## 4 Concluding Remarks

We have outlined the theoretical basis of various approaches to estimate the macrostate transition matrix of an MSM, from the standard local equilibrium approximation to a sophisticated generalized master equation approach. Employing a one-dimensional toy model and an all-atom folding trajectory of HP35, we have provided a comprehensive comparison of the various formulations, and introduced two new methods, the microstate-based approach in eq 9 and the hybrid MD/MSM ansatz in eq 14.

The most commonly used method, the local equilibrium approximation (eq 8), simply counts the transitions between the macrostates within a certain lag time, which assumes Markovian data with a time scale separation between fast intrastate fluctuations and slow interstate transitions. As shown for the toy model in Figure 1, which allows to tune the ratio between these time scales, the quality of the resulting implied time scale and the Chapman-Kolmogorov test readily deteriorate, when this separation is not fulfilled.

Assuming that Markovianity is at least given at the microstate level, the knowledge of the microstate dynamics can be exploited to construct improved macrostate dynamics. For one, this is used in the Laplace transform-based method by Hummer and Szabo,^13^ which was shown to achieve significantly improved Markovianity in all considered cases. However, we can also directly employ a microstate-to-macrostate projection to construct a macrostate transition matrix ***T***_Mic_(*t*) (eq 9), which by design yields correct macrostate population dynamics. As an important consequence, this means that residual non-Markovian behavior of the resulting MSM must be caused by suboptimal microstates (and not by the lumping of micro- into macrostates). By performing microstate Monte Carlo Markov chain simulations with subsequent projection on macrostates, this new and promising microstate-based approach may also provide state-to-state waiting times and transition pathways.

Not relying on the quality of the microstates, generalized master equations, such as the qMSM ansatz of Huang and co-workers^17^ provide an alternative approach to deal with non-Markovian dynamics. In fact, we found that qMSM performs well even in the case of ill-defined macrostates of HP35 (Figure 2) and in the case of short-trajectory data (Figure 3). We discussed several peculiarities of the approach, including the interpretation of the memory kernel matrix and its calculation from noisy data.

In practice, an MSM is often used to interpret given MD data in terms of the time evolution of the populations *P*_*I*_(*t*) of metastable conformational states. For the length of the MD trajectories *t*_max_ (actually some hundreds MD frames less to facilitate a sufficient time average), we can simply project the MD data on these states, without invoking a dynamical approximation such as the assumption of Markovianity. In the “hybrid MD/MSM” method (eq 14), we exploit this idea to obtain the system’s short time evolution with good time resolution, and subsequently use some formulation (including local equilibrium, Hummer-Szabo, or microstate-based) to construct a MSM using the long lag time *t*_max_. Given sufficiently long trajectories, this strategy of combining the best of two worlds is simple and promises prime results. While the definition of the hybrid MD/MSM method and its virtues seem obvious, it has to the best of our knowledge not yet been fully appreciated.

## Data Availability

The simulation data and all intermediate results for our reference model of HP35, including detailed descriptions to reproduce all steps of the analyses, can be downloaded from https://github.com/moldyn/HP35.

## Supporting Information Available

Eigenvectors, time scales, and kernel decay times of all considered one-dimensional models, time evolution of the memory kernel of HP35, and structural characterization of the states of the short-trajectory MSM of HP35 (PDF)
