# Supplementary material for: Markov-Type State Models to Describe Non-Markovian Dynamics
Source: J Chem Theory Comput. 2025 Feb 26;21(5):2757–65. doi: 10.1021/acs.jctc.4c01630 (PMC11912194; doi:10.1021/acs.jctc.4c01630)
Supplement: Supplementary file 1 — ct4c01630_si_001.pdf [file ct4c01630_si_001.pdf]

Supporting Information: Markov-type state models to describe non-Markovian dynamics

Sofia Sartore, Franziska Teichmann, and Gerhard Stock*

Biomolecular Dynamics, Institute of Physics, University of Freiburg, 79104 Freiburg, Germany

E-mail: stock@physik.uni-freiburg.de

Table S1: Timescales of the 4-state toy model, obtained for the three cases with different Markovianity. Shown are the three implied timescales t_k, the corresponding eigenvectors, the decay time of the memory kernel τ_k, and the initial values of the memory kernel $K(1)$.

	$\mathbf{h = 0.1, k = 0.5}$	$\mathbf{k = 0.1 = h}$	$\mathbf{h = 0.5, k = 0.1}$
t_1	10.6	16.6	10.59
t_2	0.45	4.48	4.48
t_3	0.26	2.39	0.45
eigenvectors	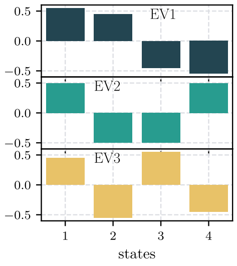	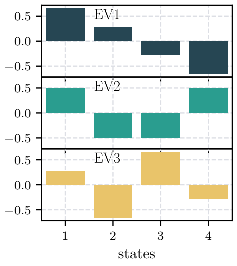	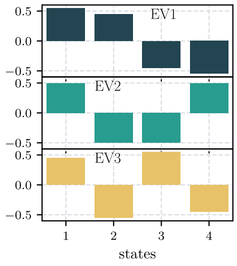
τ_k	0.45	2.8	0.83
$K(1)$	0.005	0.005	0.12

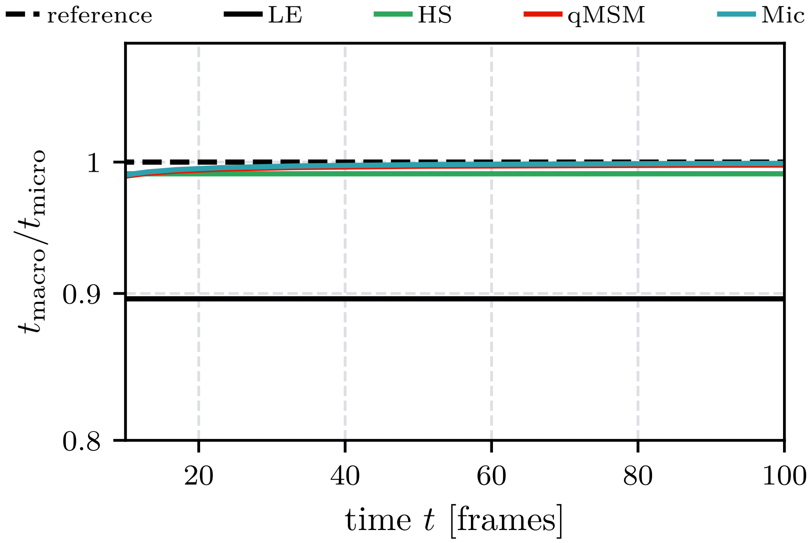

Figure S1: Implied timescale t_{macro} of the two-macrostate toy model for the Markovian case $h/k = 0.2$. Compared are the reference result obtained from the microstates (dashed black), the local equilibrium approximation (black), and the Hummer-Szabo projection (green), as well as the time evolution the result from qMSM (using the Kernel time $\tau_K = 3$, red) and of the microstate-based result (blue).

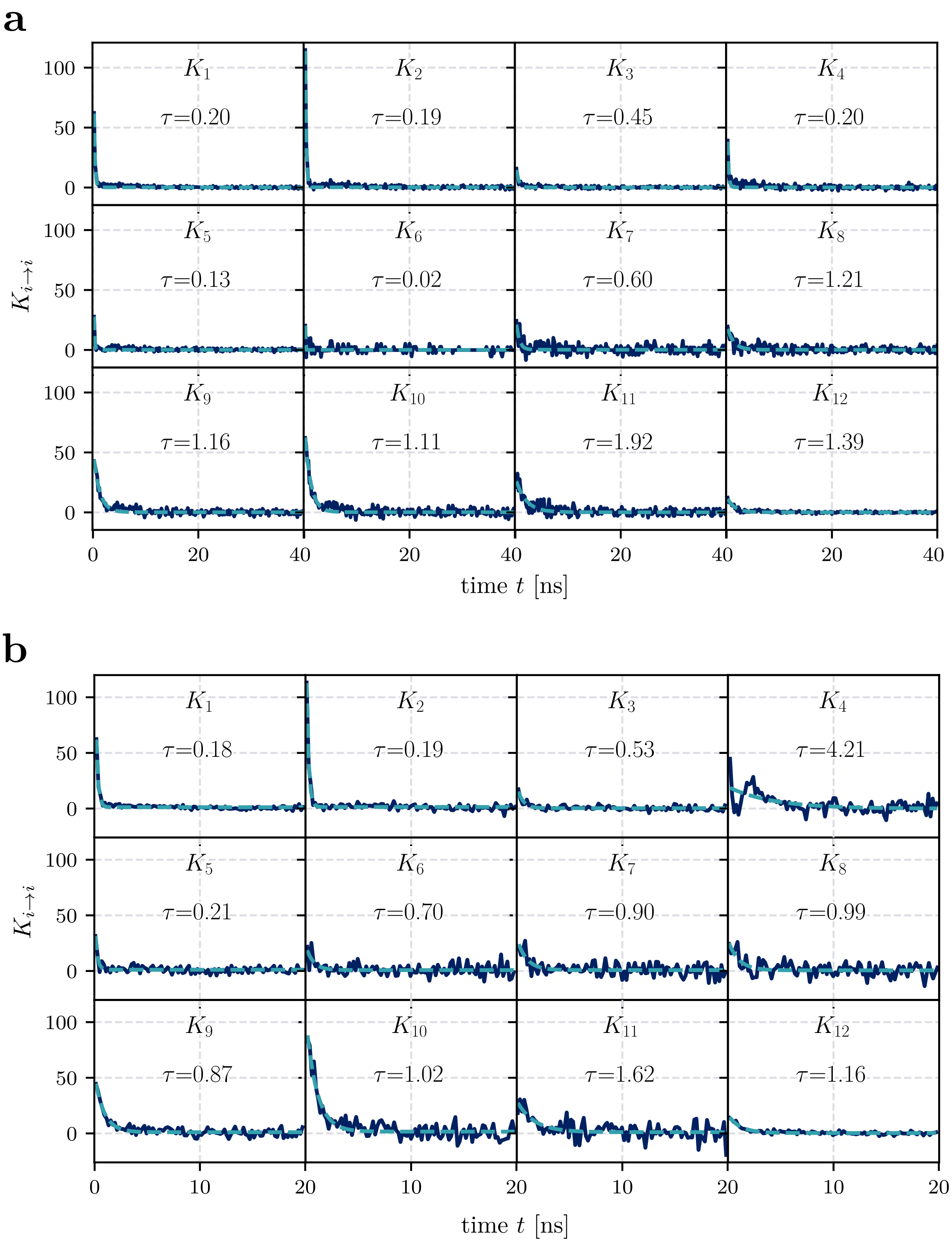

Figure S2: (a) Diagonal element of the qMSM memory kernel for the 300 μs trajectory of HP35. The kernels are each fit with an exponential function and the decay times estimated from each fit are shown. The kernels obtained from the 30 ns trajectories are quite similar, as shown in (b).

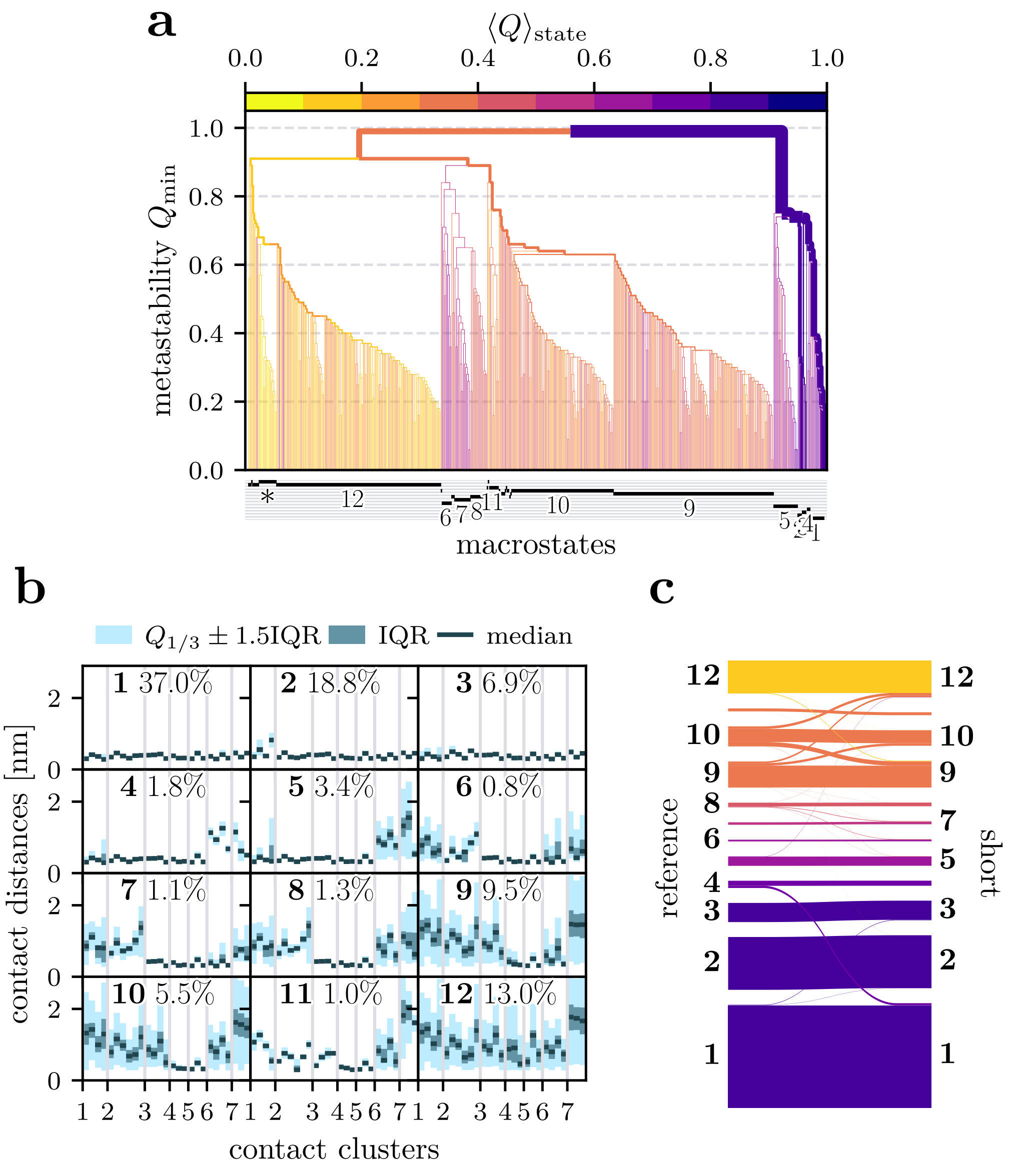

Figure S3: States analysis for the 12 states obtained by lumping the short microstates trajectories as described in Sect. III.B of the main paper. (a) MPP dendrogram[S1] demonstrating the classification of microstates into metastable states, (b) the contact representation of the resulting metastable states, (c) a Sankey diagram contrasting the states of the reference model[S2] on the left and the states from the short trajectories on the right.

References

(S1) Jain, A.; Stock, G. Identifying metastable states of folding proteins. *J. Chem. Theory Comput.* **2012**, *8*, 3810 – 3819.

(S2) Nagel, D.; Sartore, S.; Stock, G. Selecting Features for Markov Modeling: A Case Study on HP35. *J. Chem. Theory Comput.* **2023**, *19*, 3391 – 3405.
